# Dynamics of communication on measles vaccination on digital platforms in the Brazilian context: challenges and perspectives

**DOI:** 10.1590/S1678-9946202466068

**Published:** 2024-12-06

**Authors:** Maria da Penha Soares Silva, Vera Lúcia Gattás, Expedito José de Albuquerque Luna

**Affiliations:** 1 Universidade de São Paulo, Faculdade de Medicina, Instituto de Medicina Tropical de São Paulo, Programa de Pós-Graduação em Medicina Tropical, São Paulo, São Paulo, Brazil; 2 Instituto Butantan, Diretoria Médica de Ensaios Clínicos e Farmacovigilância, São Paulo, São Paulo, Brazil; 3 Universidade de São Paulo, Faculdade de Medicina, Departamento de Medicina Preventiva, São Paulo, São Paulo, Brazil

**Keywords:** Vaccination hesitancy, Social media, Disinformation, Measles, Anti-vaccination movement

## Abstract

The infodemic and the spread of disinformation have fostered mistrust in vaccines, health institutions, and governments, contributing to a global decline in vaccination coverage and the resurgence of vaccine-preventable diseases like measles. In recent years, the use of digital platforms to access health information, including vaccines, has increased significantly. However, the rapid dissemination of disinformation on these under-regulated platforms can greatly influence vaccination behavior. This study aimed to identify and analyze the main arguments used on Facebook^®^ regarding measles vaccination. Posts and comments in Brazilian Portuguese were extracted using keywords such as “Measles Vaccines,” “Triple Viral,” and “Tetra Viral” from general and anti-vaccine pages from January 2017 to December 31, 2020. A sample from both datasets was selected and analyzed using deductive content analysis. Of the posts, 213 (84.5%) were classified as pro-vaccine, primarily promoting vaccination campaigns, with limited discussions on vaccine risk-benefit and collective responsibility. Notably, anti-vaccine pages, though fewer in number and followers, were more active in posting than pro-vaccine pages. Of the anti-vaccine posts, 118 (59.3%) focused on undermining vaccine safety and efficacy, spreading disinformation to downplay disease risks. Although pro-vaccine messages showed the highest engagement on the platform, more effective communication strategies are needed to complement traditional health systems, as anti-vaccine posts appear to influence vaccination behavior, particularly among hesitant communities.

## INTRODUCTION

Vaccination is one of the most effective, safe, and cost-efficient methods for disease prevention. Despite its health benefits, a portion of the population opposes or rejects vaccines^1^. However, achieving the full benefits of herd immunity requires a high percentage of the population to be vaccinated^2^.

In 2022, World Health Organization (WHO) redefined vaccine hesitancy as a motivational state characterized by conflict or opposition to vaccination, including uncertainty regarding intent and willingness^3^. Vaccine hesitancy has raised global concern among researchers and was identified as one of the top 10 global health threats in 2019^4^.

Dubé *et al*.^5^ categorized vaccine behavior into four groups: active pro-vaccine individuals, who fully embrace vaccines due to their perceived benefits; passive pro-vaccine individuals, who follow recommendations and societal pressures; hesitant individuals, who delay or reject some vaccines; and anti-vaccine individuals, who not only refuse all vaccines but also actively advocate against vaccination, seeking to influence policies.

Vaccine hesitancy is a complex issue, encompassing a spectrum that includes individuals who reject all vaccines at one end, those who fully accept all vaccines at the other, and those in between who selectively accept some vaccines while refusing others^1^.

Several key factors contribute to vaccine hesitancy: confidence in vaccine safety and efficacy, complacency linked to the perceived risk of contracting preventable diseases, convenience in terms of vaccine access, collective responsibility for herd immunity, and calculation, where individuals weigh the risks and benefits before deciding^1,6^.

The Brazilian National Immunization Program (NIP), established in 1973 for combating vaccine preventable diseases, has also been facing challenges, with vaccination rates declining by 10% to 20% since 2016^7-9^. The COVID-19 pandemic in 2020 further exacerbated this issue. Decreased vaccination coverage has enabled the resurgence of previously controlled diseases, such as the reemergence of measles in Brazil, which was declared eliminated in 2016 but returned in 2018 and continues to spread across states^10-12^. Brazilian Ministry of Health confirmed 8,427 cases of measles from December 29, 2019 to December 26, 2020^13^.

Globally, measles outbreaks have been observed, especially in regions where the measles, mumps, and rubella (MMR) vaccine has been refused^14^. While vaccine refusal has been common in history, recent years have shown a significant rise in anti-vaccination movements (AVMs) worldwide^15^. Despite the consistency of their arguments, AVMs have gained momentum via the rapid spread of vaccine disinformation via digital platforms^15,16^.

Digital platforms like Facebook^®^ are particularly effective at disseminating information quickly and affordably, reaching wide audiences in a largely unregulated environment^17^. The infodemic and the amplification of disinformation have led to a global decline in vaccination rates, resulting in the resurgence of vaccine-preventable diseases such as measles^18,19^.

Studies indicate that digital platforms amplify disinformation via biases such as implicit bias (trust in information shared by individuals in one’s social network), confirmation bias (engaging with content that aligns with pre-existing beliefs), and repetition bias (frequent exposure to the same information within discussion groups)^20,21^.

These biases increase the perceived risks of vaccines while minimizing the perceived risks of not vaccinating^22,23^.

Approximately 5% of all internet searches pertain to health topics, emphasizing the importance of leveraging digital platforms to disseminate accurate health information. Ensuring that individuals can access, understand, and use credible health information is critical to making appropriate health decisions^24,25^.

This study aims to analyze the dynamics of communication and arguments about measles vaccination on digital platforms, specifically Facebook^®^.

## MATERIALS AND METHODS

This study employed qualitative content analysis on data collected from the digital platform Facebook^®^.

Facebook^®^ was chosen due to its prominence as one of the most widely used platforms in Brazil, with over 120 million active users. Globally, in 2019, Facebook^®^ surpassed 2 billion active users, making it one of the largest digital communities, followed by YouTube and WhatsApp^25-27^.

Posts, defined as any content shared by users on Facebook^®^ (including text, images, videos, links, or combinations thereof) in Brazilian Portuguese, were extracted using the CrowdTangle tool (List IDs: 1543055, 15502111, Facebook^®^, Menlo Park, California, USA). CrowdTangle tracks interactions such as like, shares, comments, and link clicks on public contents from Facebook^®^ pages and groups. Comments from selected posts were extracted using the Export Comments software (version 2.0.0, Export Comments Company), which extracts up to 100 comments per post from various digital platforms, including Facebook^®^, in its free version.

A total of two distinct datasets were extracted and analyzed: the first dataset focused on general Facebook^®^ pages using the keywords “Measles Vaccine,” “Triple Viral,” and “Tetra Viral” from January 2017 to December 2020. The second dataset targeted a public anti-vaccine Facebook^®^ group titled O Lado Obscuro das Vacinas (The Dark side of Vaccines), consisting of 14,901 members when the data were extracted (December 2014 to May 2021).

The analysis was conducted in three stages. First, the data were contextualized to identify the number of posts about measles vaccines, the time lapse between posts, and the groups publishing information on this topic. Second, a qualitative analysis of the posts was conducted using seven categories: (a) type of pages, (b) page description (vaccine or non-vaccine-specific content), (c) sentiment analysis, (d) presence of disinformation, (e) context of the post, (f) determinants of vaccine hesitancy, and (g) types of resources used. The third stage involved a qualitative analysis of the comments, categorizing them into anti-vaccine/vaccine hesitancy, pro-vaccine/vaccination encouragement, vaccination queries, and criticisms. The definitions of these categories and codes are detailed in the codebook (Supplementary File S1).

For this analysis, only posts with a performance score equal to or greater than zero were included. Posts with low performance (score less than or equal to zero) were excluded. The CrowdTangle tool provides this performance metric, in which a post is considered over-performing when it surpasses a benchmark based on interactions (likes, shares, comments, and link clicks) as compared to expected metrics.

Data from the posts and comments were entered into Microsoft Excel (version 2406, Redmond, Washington, USA) and subsequently imported into ATLAS.ti (version 23.1.2, ATLAS.ti GmbH, Berlin, Germany) for deductive analysis using codes and categories.

## RESULTS

### Contextualization

**Figure 1:**
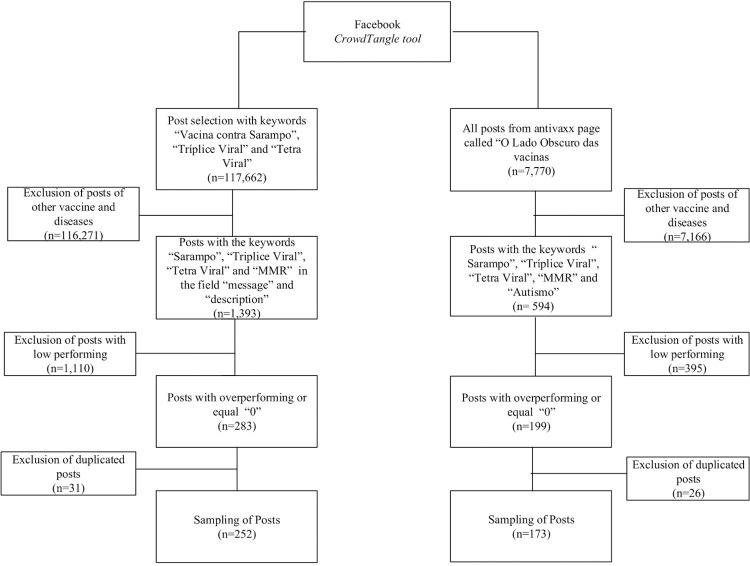
Research flowchart.

A total of 19,377 pages were analyzed, comprising 117,662 posts containing the keywords “Measles Vaccine,” “Triple Viral,” and “Tetra Viral” from January 1, 2017 to December 31, 2020. Additionally, 7,770 posts from the anti-vaccine page “The Dark Side of Vaccines” were analyzed, covering the period from its creation on December 28, 2014 to May 1, 2021. Figure 1 shows the research flowchart.

The 10 pages with the highest number of posts extracted by keyword search totaled 2,374 posts, averaging 237 posts. The average time between posts on these pages was five days during the analyzed period. In contrast, the anti-vaccine page The Dark Side of Vaccines published 5,489 posts, with an average interval of 0.3 days between them.

The page of Brazilian Society of Immunization (SBIm) showed the highest number of posts during the period, totaling 279 posts with an average time of 5.1 days between them. However, the Brazilian Ministry of Health and Ministry of Health – Vaccination pages exhibited the highest engagement, defined as any action taken on a Facebook^®^ post, page, group, or ad, including likes, shares, comments, and link clicks. These pages recorded 552,714 and 518,150 interactions, respectively, from 2017 to 2020, as shown in Table 1.

**Table 1:** Facebook® pages with post about vaccines with highest engagement from 2017 to 2020.

Number	Page name	URL Page	Share	Comments	Posts	Likes	Total Interactions
1	Ministerio da Saude (Ministry of Health)	https://www.facebook.com/minsaude	321,689	24,939	175	205,911	552,714
2	Ministerio da Saude - Vacinacao (Ministry of Health – Vaccination)	https://www.facebook.com/VacinacaoMS/	162,773	28,671	112	326,594	518,150
3	Dr. Diego Pediatra (Dr. Diego Pediatrician)	https://www.facebook.com/diegopediatria	84,619	25,873	266	115,174	225,932
4	Quebrando o Tabu (Breaking the taboo)	https://www.facebook.com/quebrandootabu	67,812	12,608	12	116,598	197,030
5	Frases 1997 (Phrases 1977)	https://www.facebook.com/profile.php?id=100067705695857	62,411	777	1	1,995	65,184
6	Sociedade Brasileira de Imunizacoes (Brazilian Society of Immunizations)	https://www.facebook.com/sbimoficial	51,112	2,847	279	88,876	143,114
7	BBC News Brasil (BBC News Brazil)	https://www.facebook.com/bbcnewsbrasil/?locale=pt_BR	50,859	9,395	70	53,006	113,330
8	G1 - Portal de noticias da Globo (G1 - Globo news portal)	https://www.facebook.com/g1?locale=pt_BR	48,024	19,905	66	88,719	156,714
9	Associacao Brasileira de Profissionais de Epidemiologia de Campo (Brazilian Association of Field Epidemiology Professionals)	https://www.facebook.com/AssociacaoProEpi?locale=pt_BR	46,574	232	132	4,655	51,593
10	HISTORY	https://www.facebook.com/CanalHISTORY?locale=pt_BR	45,968	4,411	6	18,111	68,496
11	SAD	https://www.facebook.com/S4D2022?locale=pt_BR	45,690	808	1	10,278	56,777
12	Valorizacao Medica - 2 (Medical Appreciation – 2)	https://www.facebook.com/valorizacaomedicarj?locale=pt_BR	35,583	18	5	440	36,046
13	Jornal O Globo (Newspaper O Globo)	https://www.facebook.com/jornaloglobo?locale=pt_BR	32,937	10,500	72	35,263	78,772
14	Jornal Extra (Newspaper Extra)	https://www.facebook.com/jornalextra?locale=pt_BR	28,723	4,089	36	26,589	59,437
15	Minha Vida (My Life)	https://www.facebook.com/minhavida?locale=pt_BR	28,671	2,540	56	14,728	45,995
16	Prefeitura da Cidade de Sao Paulo (São Paulo city administration)	https://www.facebook.com/PrefSP?locale=pt_BR	28,280	5,739	57	100,145	134,221
17	Silas Malafaia	https://www.facebook.com/SilasMalafaia?locale=pt_BR	28,231	3,693	1	24,132	56,057
18	Enfermeira Ironica (Ironic Nurse)	https://www.facebook.com/enfermeiraironica?locale=pt_BR	27,932	1,057	15	12,060	41,064
19	Secretaria Municipal da Saude de Sao Paulo (Sao Paulo Municipal Health Department)	https://www.facebook.com/saudeprefsp?locale=pt_BR	26,177	4,916	116	11,085	42,294
20	Portal R7	https://www.facebook.com/portalr7?locale=pt_BR	23,347	11,682	121	57,350	92,500
**Total**			**1,247,412**	**174,700**	**1,599**	**1,311,709**	**2,735,420**

### Posts analysis

A total of 252 posts were selected from the general Facebook^®^ pages using keyword search, along with 173 posts from the anti-vaccine page dataset, based on the criteria outlined in Figure 1 for deductive qualitative analysis.

#### Type of page where the posts were published

The dataset analysis revealed that most pages publishing posts about measles vaccines were news content pages (32.9%).

## Page description

The analysis of page descriptions showed that only 8.7% of the pages featured at least 10 consecutive posts dedicated to vaccine-related content. This suggests that most pages were not primarily focused on vaccine awareness, with publications being occasional or sporadic.

## Sentiment analysis

The sentiment analysis of keyword search data revealed that 84.5% of posts held a pro-vaccine stance. As expected, the anti-vaccine dataset showed a predominant presence of content against vaccination or related to vaccine hesitancy (Table 2).

**Table 2:** Posts sentiment analysis.

Sentiment analysis	Keyword search		Antivaccine page	
	n	%	n	%
Pro-vaccine	213	84.5	2	1.2
Neutral	32	12.7	35	20.2
Antivaccine/Hesitant	7	2.8	136	78.6
Total	252	100	173	100

## Disinformation

Disinformation was identified in 66.5% of posts on the anti-vaccine page and in posts with anti-vaccine content from the keyword search dataset. These posts often misrepresented scientific studies, media reports, and case reports of alleged vaccine-related adverse events. Conspiracy theories were also prevalent, targeting the credibility of health institutions, governments, the media, and the pharmaceutical industry (Table 3).

**Table 3:** Posts with disinformation.

Disinformation	Keyword search		Antivaccine page	
	n	%	n	%
No	246	97.6	58	33.5
Yes	6	2.4	115	66.5
Total	252	100	173	100

## Context of posts’ arguments

Of the posts extracted via keyword search, 55.6% aimed to promote vaccination campaigns, with over 80% focusing on providing information about the locations and dates of the campaigns, emphasizing vaccine accessibility. The second most discussed topic was disease risk perception (7.1%), often including details about the resurgence of the disease and its symptoms and transmission. On the anti-vaccine page, posts were primarily concerned with vaccine safety and efficacy (59.3%), followed by conspiracy theories (13.6%) and civil liberties (11.6%). Table 4 summarizes the identified contexts.

**Table 4:** Context of posts’ arguments*.

Context of posts’ arguments	Keyword search		Antivaccine page	
	n	%	n	%
Vaccination campaign promotion	140	55.6	0	0
Vaccine safety and efficacy	98	38.9	118	59.3
Other context	7	2.7	10	5.0
Political and government	3	1.2	9	4.5
Conspiracy theory	2	0.8	27	13.6
Civil liberty	2	0.8	23	11.6
Alternative treatment	0	0	7	3.5
Religion and morality	0	0	5	2.5
Total	252	100	199	100

*Some posts from the antivaccine page were coded in more than one category, totaling 199 codes, which is more than the total number of posts (173).

The primary argument on anti-vaccine pages revolved around vaccine safety, often citing associations with autism, neurological diseases, and weakened immune systems due to preservatives such as aluminum and mercury/thimerosal. The following posts illustrate the context of safety and efficacy associated with disinformation on the anti-vaccine page.

“Yes, there is a huge amount of scientific evidence that conclusively and convincingly determines the irrefutable fact that mercury is toxic when injected into the bodies of children via vaccines” (Post 107:9 - Page: The Dark Side of Vaccines; our translation)

“I believe that the large number of children with Autism, ADHD, ODD, etc., today is related to the amount of vaccines (full of heavy metals) given to children currently compared to about 15 years ago, which were very few. The package insert of some even states that one of the side effects is Autism. What do you think?” (Post 147:3 - Page: The Dark Side of Vaccines; our translation)

## Determinant of vaccine hesitancy

In the keyword search dataset, convenience emerged as the most frequently mentioned determinant in pro-vaccine posts, particularly in the context of campaign promotion. In contrast, anti-vaccine posts primarily focused on undermining vaccine safety and diminishing public trust. Table 5 shows the frequency of determinants mentioned in the posts.

**Table 5:** Determinants of vaccine hesitancy.

Determinants of vaccine hesitancy	Keyword search		Antivaccine page	
	n	%	n	%
Convenience	148	58.7	1	0.6
Confidence	43	17.1	127	73.4
Complacency	41	16.3	29	16.7
Collective responsibility	16	6.3	1	0.6
Unclassifiable	3	1.2	13	7.5
Calculation	1	0.4	2	1.2
Total	252	100	173	100

## Type of resource used in the posts

The anti-vaccine page predominantly used text (39.3%) in its posts, with many featuring lengthy translations of content from other countries, mainly the United States. In contrast, pro-vaccine posts, identified via the keyword search, primarily used images (60.7%).

## Posts’ comments analysis

A total of 801 comments were analyzed, with 659 comments associated with 12 pro-vaccine posts and 142 comments linked to six anti-vaccine posts. Most comments were posted from one to 50 days after the post date, with a mean time of five days. Peak interaction typically occurred within the first two days after the post.

Among pro-vaccine comments, 443 (67.2%) promoted vaccination, whereas 108 (76.1%) of the comments on anti-vaccine posts expressed anti-vaccine sentiments or vaccine hesitancy. Pro-vaccine posts often contained calls to action, in which users mentioned the names of individuals or groups to increase awareness of vaccination campaigns. However, discussions around the risks and benefits of vaccination, as well as collective responsibility, were less common in the comments.

There is a noticeable polarization in discussions about vaccination, with opposing arguments often refuted. The following user comment is an example.

“Anyway, if you’re in favor of vaccines that do much more harm than good to everyone... You’re in the wrong group. Here, people really know what vaccines are in society, what they’re for. And what they cause. If you don’t know, I ask you to study the entire composition of vaccines and side effects. Don’t believe that vaccines immunize. That’s a lie.”

Another user commented on the difficulty of dialogue between pro-vaccine and anti-vaccine groups.

“I think it’s healthy to have a discussion for and against vaccines, the problem is they don’t even want to entertain the possibility of dialogue. I went to talk to a biologist friend, ask a few things humbly, and he compared me to flat-earthers.”

Queries about age groups, target audiences, and contraindications were common in 92 (13.96%) pro-vaccine posts, especially those related to campaign promotion during disease outbreaks. Many of these inquiries remained unaddressed by the post authors, with limited guidance provided to those seeking information.

## DISCUSSION

Measles vaccination was a prominent topic on digital platforms during 2018 and 2019, driven by the resurgence of the disease in Brazil. While pro-vaccine pages, such as the Brazilian Society of Immunization (SBIm), posted more frequently overall and the Brazilian Ministry of Health pages showed higher engagement, anti-vaccine posts demonstrated greater influence due to their higher posting frequency and the emotional nature of their contents. Anti-vaccine pages, despite showing fewer followers, posted much more frequently (0.3-day intervals compared to 5.1-day intervals for pro-vaccine pages), which led their messages, often centered around disinformation on vaccine safety and efficacy, to remain highly visible and continuously engage their audience. This trend supports prior research, indicating that anti-vaccine individuals use digital platforms more continuously and frequently, while pro-vaccine individuals tend to become more active primarily during outbreak periods^28,29^.

Since 2020, a decline in measles vaccination posts has been noted, likely due to the COVID-19 pandemic and increased efforts to regulate anti-vaccine content on digital platforms^30^. This reduction is concerning, given the rising use of digital channels for health information dissemination and the increase in measles cases across Brazilian states^29,31,32^.

Interestingly, most vaccine-related discussions occurred on pages not primarily focused on health, highlighting the need for a more proactive presence from health institutions. Though the Brazilian Ministry of Health pages showed high engagement, their lower posting frequency suggests that their pro-vaccine messages may have reached fewer users. Brazilian Society of Immunization (SBIm) effectively disseminated educational content while also serving as a reliable source for news and press page.

The limited engagement by technical-scientific societies and health institutions on digital platforms may contribute to the disproportionate influence of anti-vaccine content, as these emotionally charged and conspiratorial narratives become more effective in shaping public perception, particularly among vaccine-hesitant individuals. Investing in consistent, targeted pro-vaccine communication strategies, including direct public engagement via trained digital influencers, is essential to counter disinformation and build trust, especially in vaccine-hesitant communities^33-35^.

During the study period, pro-vaccine posts predominantly focused on promoting vaccination campaigns in response to disease outbreaks. However, there was limited discussion around collective responsibility and the evaluation of the risks and benefits of vaccination^36^.

A clear polarization was observed in posts and comments, with a limited constructive dialogue between anti-vaccine or hesitant communities and those who are pro-vaccine, which is aligned with previous studies^5^. Pro-vaccine posts often portrayed vaccine hesitancy as stemming from ignorance, while anti-vaccine posts framed the uncritical acceptance of vaccines as societal alienation. These findings underscore the role of digital platforms in fostering echo chambers, reinforcing biases, and solidifying truth perceptions, which discourages open debate on vaccination^34-35,37^. Additionally, vaccine-hesitant individuals frequently sought validation for their views on anti-vaccine pages^38^, highlighting the potential influence of digital platforms in shaping vaccination behavior via disinformation.

It is crucial to distinguish between anti-vaccine sentiment and vaccine hesitancy. Anti-vaccine groups reject vaccines outright, often based on arguments that contradict scientific evidence. In contrast, vaccine-hesitant individuals express legitimate concerns about safety and efficacy, typically influenced by disinformation. A lack of accurate information and low health literacy further contribute to vaccine hesitancy, especially among those seeking more data to make informed decisions.

The results of this study emphasize vaccine selection behavior, with a belief that some vaccines are essential, while others are deemed unnecessary.

The limited presence of pro-vaccine digital influencers highlights the need to invest in training communicators who can deliver impactful, credible messages. Health communication on digital platforms is evolving, and underestimating the public’s ability to comprehend the risk-benefit analysis of vaccines allows anti-vaccine activists to gain influence.

The analysis of comments on pro-vaccine posts, particularly those promoting campaigns, frequently included inquiries about target audiences and contraindications. Notably, the absence of curation to address these queries may prompt individuals to seek information from less reliable sources, exacerbating the spread of vaccine-related disinformation.

In the context of anti-vaccine posts, a significant and frequent activity was observed. These posts often emphasized vaccine safety concerns, associating vaccines with risks such as autism, neurological disorders, and reduced immune system capacity. Claims regarding harmful substances like mercury and aluminum in vaccines were also prevalent^36,39^. Disinformation within these posts distorted scientific data, fueling distrust in health institutions^40^.

Vaccine hesitancy is a complex issue, and arguments for or against vaccination may vary depending on context, timing, and the specific vaccine in question. Although this study focused on measles vaccines, the results align with previous research on vaccine hesitancy and refusal on digital platforms. Future research should explore vaccine hesitancy related to other vaccines, particularly COVID-19 vaccines, which have been widely administered globally. These findings contribute to the academic discussion on the impact of digital platforms on vaccination adherence in Brazil.

This study was limited to data from a single digital platform, which, although one of the most widely used in Brazil, may not capture the dynamics of other significant digital media such as YouTube, Twitter, and Instagram. Data extraction was conducted using CrowdTangle, which only enabled the collection of public posts. As a result, data from private groups were not analyzed; however, given the smaller number of private groups compared to public ones, this limitation likely had minimal impact on the overall results.

The selected anti-vaccine page was removed from Facebook^®^ in 2022, limiting the scope of comment extraction. However, the comments extracted before the exclusion were analyzed. Digital platforms are dynamic environments, and retrospective research can be challenging, as content may be removed or made private, potentially impacting study outcomes depending on the period and keywords used.

Few studies address the causes of vaccine hesitancy in Brazil, and even fewer examine this issue on digital platforms. Future research should explore the arguments for and against vaccination across various digital platforms, particularly investigating the influence of anti-vaccine arguments on vaccination decisions. Additionally, exploring the role of instant messaging apps such as WhatsApp and Telegram in shaping vaccination behavior is essential. The methodology used in this study can aid further understand the role of digital platforms in discussions on other vaccines, especially COVID-19 vaccines.

## CONCLUSION

This study sheds light on the complexities surrounding vaccine hesitancy and declining vaccination coverage, challenging the common narrative that attributes these phenomena solely to anti-vaccine movements. While these movements play a significant role in spreading disinformation, the results indicate other critical factors that require attention.

As digital platforms increasingly serve as key sources of health information, health and governmental institutions must develop effective communication strategies to complement traditional systems. Continuous monitoring of digital platforms is essential for identifying arguments and resources that generate higher engagement. Policies aimed at combating disinformation and fostering vaccine confidence should be based on these analyses.

Relying solely on communication during outbreaks and vaccination campaigns is insufficient to stimulate widespread vaccine uptake. The persistent threats of infodemic and disinformation underscore the need for reliable communication channels to address public insecurities. Digital platforms can play a powerful role in assessing public sentiment, allowing for more targeted, educational responses.

This research highlights the importance of more frequent and active pro-vaccine communication from medical-scientific societies, health institutions, and digital influencers. Engaging directly with the public to clarify doubts, encourage dialogue, and promote social responsibility is critical to combating disinformation and building trust in vaccines.

In conclusion, leveraging digital platforms to monitor trends and respond effectively to pro-vaccine and hesitant communities is crucial. Collaboration between health authorities, digital influencers, and the public is essential to address current and future vaccination challenges.

## Data Availability

available from: https://doi.org/10.48331/scielodata.VQ4ZHW
